# Taking a Stand Against Air Pollution – The Impact on Cardiovascular Disease: A Joint Opinion from the World Heart Federation, American College of Cardiology, American Heart Association, and the European Society of Cardiology

**DOI:** 10.5334/gh.948

**Published:** 2021-01-28

**Authors:** Michael Brauer, Barbara Casadei, Robert A. Harrington, Richard Kovacs, Karen Sliwa, the WHF Air Pollution Expert Group

**Affiliations:** 1 World Heart Federation Air Pollution Expert Group, School of Population and Public Health, The University of British Columbia, CA; 2 Institute for Health Metrics and Evaluation, University of Washington, US; 3 European Society of Cardiology, Radcliffe Department of Medicine, University of Oxford, GB; 4 American Heart Association, Department of Medicine, Stanford University, US; 5 American College of Cardiology, Indiana University School of Medicine, US; 6 World Heart Federation, Hatter Institute for Cardiovascular Research in Africa, Department of Medicine, University of Cape Town, ZA; * Prof Michael Brauer, The University of British Columbia, Institute for Health Metrics and Evaluation (University of Washington) (Canada/United States); Dr Narantuya Davaakhuu, National Center for Public Health Mongolia (Mongolia); Dr Michael Hadley, Mount Sinai (United States); Mr Daniel Kass, Vital Strategies (United States); Prof Mark Miller, Centre for Cardiovascular Sciences, University of Edinburgh (United Kingdom); Prof Maria Consuelo Escamilla Nuñez, Instituto Nacional de Salud Pública (Mexico); Prof Dorairaj Prabhakaran, Public Health Foundation India (India); Dr Ta-Chen Su, Department of Environmental and Occupational Medicine, National Taiwan University College of Medicine (Taiwan); Dr Ilonca C.H. Vaartjes, Julius Center for Health Sciences and Primary Care, University Medical Center Utrecht (The Netherlands); Dr Rajesh Vedanthan, Mount Sinai (United States)

**Keywords:** air pollution, cardiovascular disease, cvd, environmental health impacts, climate

## Abstract

Although the attention of the world and the global health community specifically is deservedly focused on the COVID-19 pandemic, other determinants of health continue to have large impacts and may also interact with COVID-19. Air pollution is one crucial example. Established evidence from other respiratory viruses and emerging evidence for COVID-19 specifically indicates that air pollution alters respiratory defense mechanisms leading to worsened infection severity. Air pollution also contributes to co-morbidities that are known to worsen outcomes amongst those infected with COVID-19, and air pollution may also enhance infection transmission due to its impact on more frequent coughing. Yet despite the massive disruption of the COVID-19 pandemic, there are reasons for optimism: broad societal lockdowns have shown us a glimpse of what a future with strong air pollution measures could yield. Thus, the urgency to combat air pollution is not diminished, but instead heightened in the context of the pandemic.

## Problem

**Figure 1:**
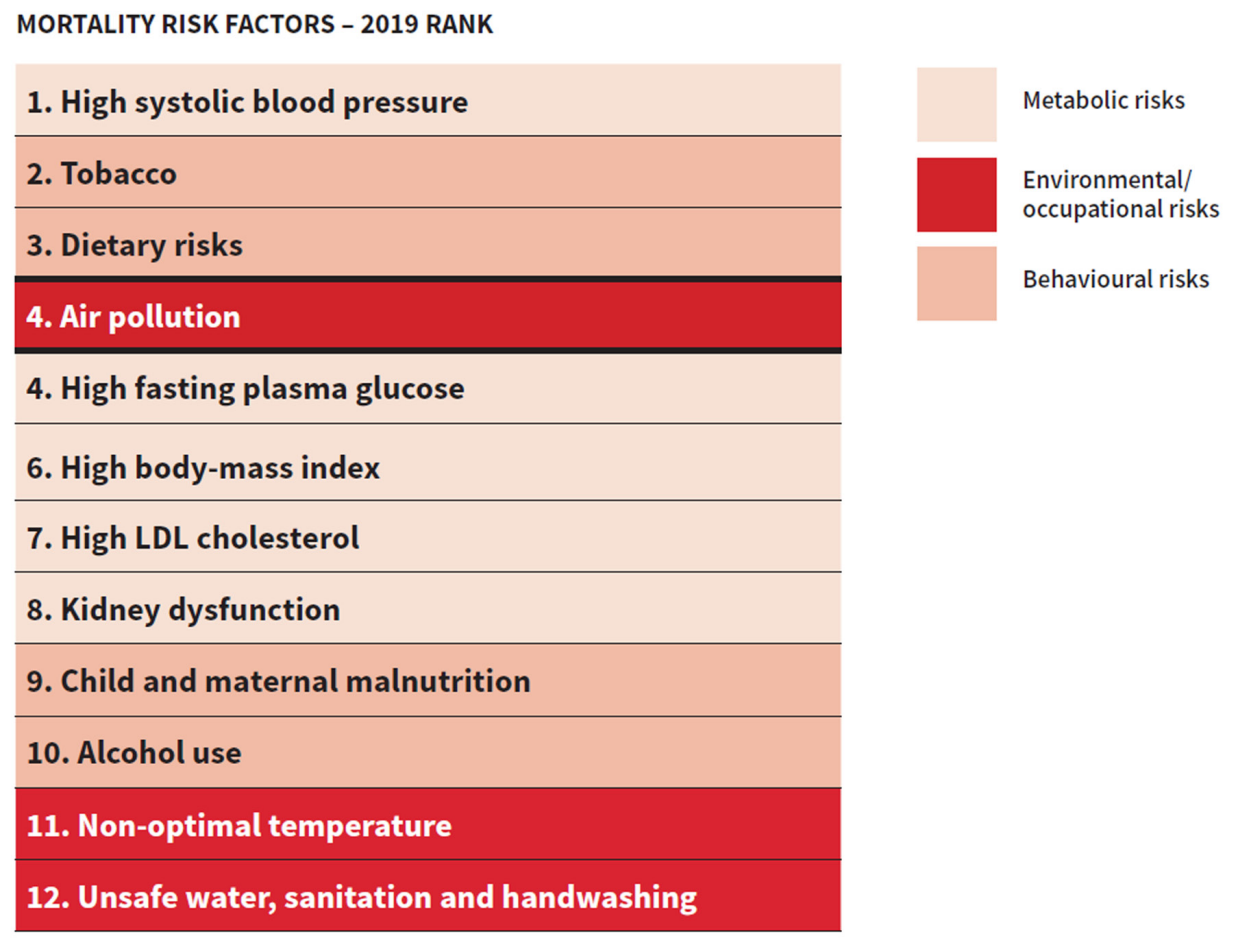
**Ranking of air pollution relative to other leading risk factors for global mortality.** Mortality Risk Factors, Both sexes, all ages, 2019. Institute for Health Metrics and Evaluation. (Adapted from Institute for Health Metrics and Evaluation, 2020).

Air pollution is a major contributor to the global burden of disease, with an estimated 12% of all deaths in 2019^1^ attributable to outdoor and household^2^ air pollution [1]. While the impacts of air pollution on respiratory diseases is widely recognized, 50% of the estimated 6.7 million deaths attributable to air pollution in 2019 are due to cardiovascular diseases [1]. Globally, nearly 20% of cardiovascular disease deaths were attributable to air pollution. Further, air pollution was the 4^th^ highest ranking risk factor for mortality, with more attributable deaths than high LDL cholesterol, high body-mass index, physical inactivity, or alcohol use (Figure 1).

Globally air pollution contributes to an average loss of life expectancy of 20 months, nearly as high as that for tobacco use (22 months), with losses of two point five years in South Asia [2]. Despite increasing awareness of the impact of air pollution on population health, appreciation of air pollution as a modifiable risk factor is still limited amongst health care providers. With this joint opinion, and in accordance with the Sustainable Development Goals, the WHF, AHA, ESC and ACC call for action in research and policy development at a global scale. Further, we aim to generate awareness about the impact of air pollution on cardiovascular disease as a means towards reaching the World Heart Federation Goal of a 25% reduction in premature cardiovascular mortality by 2025.

## Air pollution and CVD – evidence

Air pollution is a complex and dynamic mixture of numerous compounds in gaseous and particle form, originating from diverse sources, subject to atmospheric transformation and varying over space and time. Three common air pollutants, particulate matter (PM), ozone and nitrogen dioxide (NO_2_), are the focus of most monitoring programs, communication efforts [3], health impact assessments, and regulatory efforts.

Evidence for impacts on cardiovascular disease is most consistent for PM, which is responsible for the vast majority of the disease burden via its impacts on ischemic heart disease [4,5,6], and stroke [7], as well as lung cancer, COPD, lower respiratory infections, Type 2 diabetes, pregnancy outcomes and related infant mortality [8]. Time series studies conducted in hundreds of urban areas globally indicate a consistent association between short-term variability in PM and cardiovascular disease deaths [9], while large cohort studies from both high and lower income settings demonstrate increased cardiovascular disease incidence and mortality in association with PM levels [10]. Further, PM air pollution has been associated with progression of atherosclerosis [11]. Ozone is mainly associated with exacerbation of respiratory disease, with COPD incidence and mortality and with metabolic effects. NO_2_ is often used as an indicator of traffic-related air pollution. Chronic exposure to NO_2_ is associated with incident childhood asthma while short-term variability is associated with exacerbation of asthma and increased daily mortality counts.

**Figure 2:**
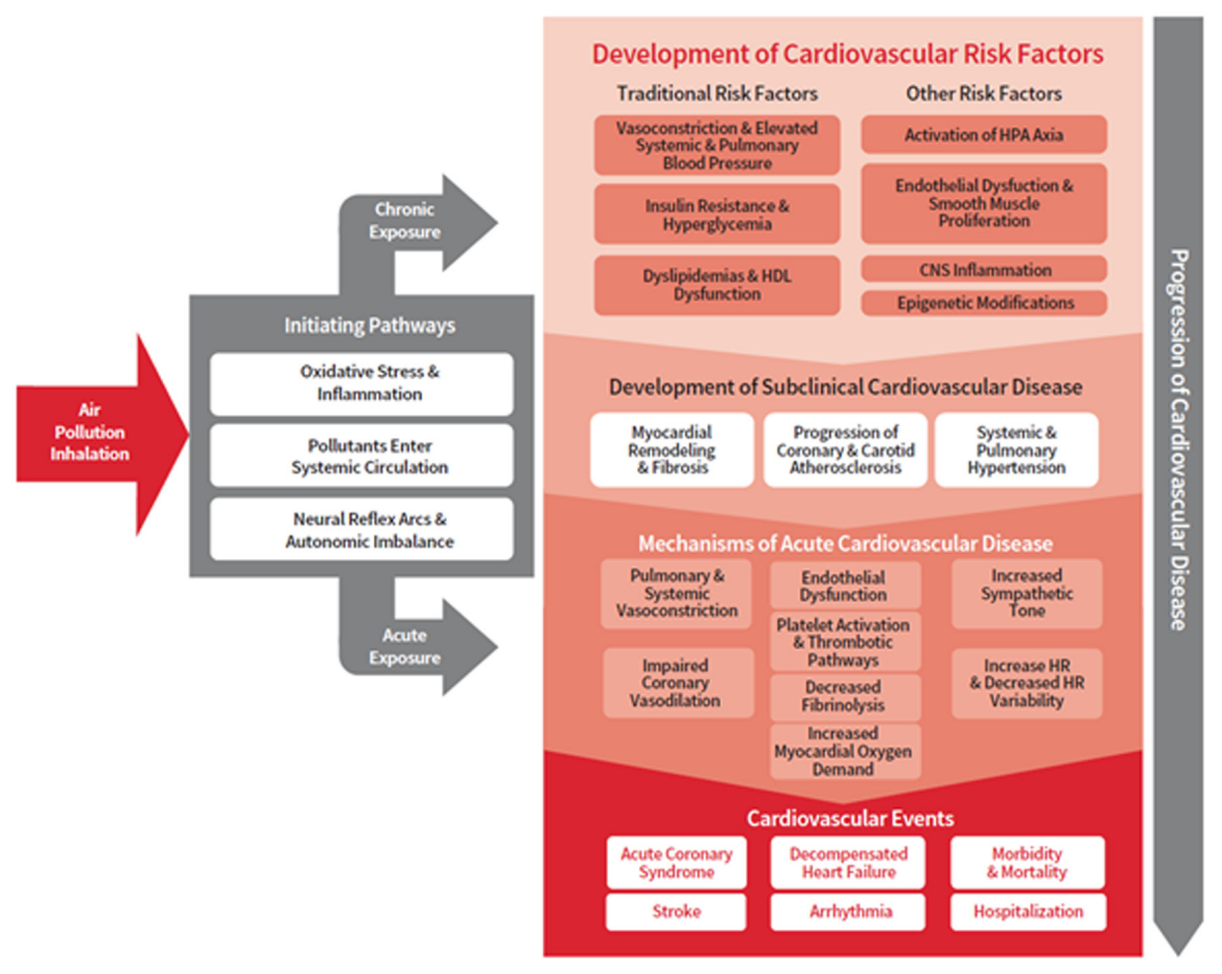
**Biological pathways linking air pollution with cardiovascular disease.** Mechanisms of cardiovascular disease attributable to air pollution exposures. (Adapted from Rajesh Vedanthan and Michael Hadley, 2019).

A Scientific Statement from the American Heart Association provides a detailed description of the pathophysiologic mechanisms through which PM triggers cardiovascular events [4]. Figure 2 summarises the likely dominant pathways including activation of oxidative stress/inflammation and autonomic imbalance, as well as translocation of components of the PM mixture (ultrafine particles or specific constituents) into the systemic circulation [4]. In turn, these alterations promote both subclinical cardiovascular disease (myocardial remodelling, atherosclerosis progression, systemic and pulmonary hypertension, enhanced vasoconstriction and coagulation) and thrombotic and non-thrombotic acute cardiovascular events (acute coronary syndromes, decompensated heart failure, stroke, life-threatening arrhythmias) [4,5,12]. The AHA statement suggests the existing evidence is consistent with a causal relationship between PM exposure and cardiovascular morbidity and mortality.

More recently, the European Society of Cardiology expert position paper updated the observational and mechanistic evidence and identified air pollution as one of several major modifiable risk factors relevant to the prevention and management of cardiovascular disease [5]. The ESC paper identified a need for additional research regarding the role of air pollution in relation to hypertension and incident heart failure. This position paper also highlighted the role of health professionals, including cardiologists, in counselling their patients on the risks of air pollution while also supporting educational and policy initiatives to reduce air pollution exposure.

## Opportunity for global impact

The threats posed by air pollution are substantial. Approximately seven billion persons (92% of the global population), live in areas where the WHO Annual Air Quality Guideline for PM_2.5_ is exceeded and three point six billion people (47% of the global population) are exposed to household air pollution from the use of solid fuels for cooking. Recent research also indicates that air pollution impacts cardiovascular health even at very low levels below current WHO guidelines and most national standards [13]. Further, climate change is leading to increased frequency and severity of wildfires, which leads to large smoke episodes and associated health impacts affecting major metropolitan areas [14].

Despite these challenges, given its near-ubiquitous exposure, air pollution reduction offers a powerful opportunity to equitably reduce cardiovascular disease. Experience also indicates a clear path forward. In the US, reductions in PM concentrations resulting from a diverse array of regulatory actions and technologies were responsible for up to 15% of the increase in US life expectancy observed over the past several decades [15,16]. Similarly, programs such as India’s Pradhan Mantri Ujjwala Yojana have made clean cooking fuels accessible to millions of households [17], helping to reduce inequalities in non-communicable diseases.

## Addressing the challenge: The role of cardiac organizations, societies, and foundations

The WHF, ACC, AHA, and ESC are united in our commitment to research, advocacy, and education to reduce the impacts of air pollution on cardiovascular health. Specifically, we will advocate for **further research on cardiovascular disease and air quality, disseminating findings** to our memberships and via other activities, such as World Heart Day. We will also work to **educate and raise awareness** among health care providers on the importance of reducing air pollution and the cardiovascular benefits of air pollution mitigation. We will work with senior decision-makers in national, regional and global governmental institutions to make air pollution related heart disease a priority and to identify interventions to reduce air pollution and its impact on NCDs. Finally, we will work with our members to increase the development and use of clinical guidelines on air pollution and cardiovascular disease to **ensure clinicians are educated** on the topic. In addition, we will strive to provide presentations on the links between air pollution and cardiovascular disease at our respective congresses, engagements, and events.

Structural actions to mitigate pollution emissions are ultimately necessary to reduce harmful exposures. Before mitigation is achieved, health care providers can play several important roles. First, clinicians can advocate for air pollution mitigation as a health measure. Second, clinicians can provide patients with personal measures to reduce exposures and associated risk at the individual level. For example, use of room air filtration can provide substantial improvements in PM levels within residences, schools and workplaces, with some evidence indicating improvements in blood pressure and measures of inflammation [18]. Third, health care providers can integrate air pollution into disease management approaches. Communication tools such as various air quality indices focused on short-term variation in air quality can help patients adjust activities when air quality is poor [19]. Detailed air pollution maps that provide information on long-term exposures and their impacts at neighborhood scales can also be used to target CVD treatment [20], exposure reduction, and efforts to address other behavioural risks contributing to CVD in those living in locations with highest exposures [21]. Finally, the health sector as a whole, which bears the impact of air pollution, can provide much-needed support for ministries of environment, energy, and transportation, which are traditionally responsible for mitigation efforts.

## Footnotes

While other estimates exist and each have their uncertainties, all estimates of attributable disease burden are large and high-ranking in comparison to traditional and more widely recognized CVD risk factors.

Household air pollution is mainly a concern in low income countries where polluting fuels (coal, wood, agricultural residue, animal dung) are used for cooking and heating.
